# A powerful on line ABTS^+^–CE-DAD method to screen and quantify major antioxidants for quality control of Shuxuening Injection

**DOI:** 10.1038/s41598-018-23748-x

**Published:** 2018-04-03

**Authors:** Huifen Ma, Jin Li, Mingrui An, Xiu-mei Gao, Yan-xu Chang

**Affiliations:** 1 Tianjin State Key Laboratory of Modern Chinese Medicine, Tianjin University of Traditional Chinese Medicine, Tianjin, 300193 China; 2 Key Laboratory of Formula of Traditional Chinese Medicine (Tianjin University of Traditional Chinese Medicine), Ministry of Education, Tianjin, 300193 China; 3 Department of Surgery, University of Michigan, Ann Arbor, MI 48109 USA

## Abstract

A novel method of on-line 2,2′-Azinobis-(3-ethylbenzthiazoline-6-sulphonate)-Capillary Electrophoresis-Diode Array Detector (on-line ABTS^+^-CE-DAD) was developed to screen the major antioxidants from complex herbal medicines. ABTS^+^, one of well-known oxygen free radicals was firstly integrated into the capillary. For simultaneously detecting and separating ABTS^+^ and chemical components of herb medicines, some conditions were optimized. The on-line ABTS^+^-CE-DAD method has successfully been used to screen the main antioxidants from Shuxuening injection (SI), an herbal medicines injection. Under the optimum conditions, nine ingredients of SI including clitorin, rutin, isoquercitrin, Quercetin-3-O-D-glucosyl]-(1-2)-L-rhamnoside, kaempferol-3-O-rutinoside, kaempferol-7-O-β-D-glucopyranoside, apigenin-7-O-Glucoside, quercetin-3-O-[2-O-(6-O-p-hydroxyl-E-coumaroyl)-D-glucosyl]-(1-2)-L-rhamnoside, 3-O-{2-O-[6-O-(p-hydroxyl-E-coumaroyl)-glucosyl]}-(1-2) rhamnosyl kaempfero were separated and identified as the major antioxidants. There is a linear relationship between the total amount of major antioxidants and total antioxidative activity of SI with a linear correlation coefficient of 0.9456. All the Relative standard deviations of recovery, precision and stability were below 7.5%. Based on these results, these nine ingredients could be selected as combinatorial markers to evaluate quality control of SI. It was concluded that on-line ABTS^+^-CE-DAD method was a simple, reliable and powerful tool to screen and quantify active ingredients for evaluating quality of herbal medicines.

## Introduction

Scientists increasingly believe that reactive oxygen species (ROS) including free radicals damage the cellular constitutive elements, especially lipids and DNA in recent years^1,2^. ROS could cause serious pathology consequences such as cell death, carcinogenesis, and premature aging^3,4^. Therefore, the antioxidants play an essential role in prevention of aging-related diseases, various cardiovascular diseases, cancer, neurological degeneration, aging and so on. Natural products, particularly herbal medicine, have been proven to have multitudinous antioxidants^5–9^. Some herbal medicines exert a therapeutic effect through the pathways of antioxidant. Thus, it is necessary to screen antioxidants for clarifying the work mechanism and ensuring the quality of herbal medicines.

Ginkgo biloba, one of famous herbal medicines with antioxidative activity^9^, is widely applied to treating geriatric diseases^10–14^. Shuxuening injection (SI) made from the aqueous extract of Ginkgo biloba also has antioxidative property. At present, SI becomes one of the most popular herbal medicine injections used for curing coronary heart disease, stenocardia and cerebral vasospasm^15–17^. For the safety of drug use, the quality of SI should be monitored comprehensively. Although a number of approaches, such as LC^18^, LC-MS^19^ and GC-MS^20^, have been developed for measuring the contents of multiple ingredients in traditional Chinese medicine (TCMs), few study of SI has been reported. LC-MS was used to measure the content of various active ingredients in previous studies^21^, but this method cannot comprehensively evaluate the pharmacological effects and quality of SI.

It was reported an on-line HPLC-DAD-CL method for evaluating the quality of Ginkgo biloba leaves based on their antioxidative activity^22^. Suppose that this developed method is applied for analyzing SI, we have to face the fact that the approach is time-consuming and requires a large amount of organic reagents. Capillary electrophoresis (CE), as a modern analytical technique, is widely used in many fields including pharmaceutical analysis^23^, screening bioactivity and quality control of natural products because of its advantages, such as easy operation, short analysis time, high efficiency and low consumption of organic reagents and sample^24–28^. The numerous researches on pharmacological activity of TCMs have been reported according to the review published previously^29^. For example, Dubber and Kanfer applied reverse-flow micellar electrokinetic chromatography for the qualitative determination of flavonols and terpene trilactones in Ginkgo biloba^30^. In this method, the amount of organic reagent decreased, but the important antioxidative activity of Ginkgo biloba could not be reflected. An activity-integrated on-line 2,2-diphenyl-1-picrylhydrazyl (DPPH)-CE-DAD method was developed for comprehensively assessing antioxidative property and quality of Reduning injection in our previous research^24^. The approach integrating DPPH with CE for measuring the total antioxidative activity of Reduning injection and the content of antioxidants in it resolved the problem of measuring activity, but the conditions of separating antioxidants and measuring the total antioxidative activity were different. Taking these facts mentioned above into account, it is essential to develop a simple, rapid and reliable method for comprehensively evaluating the pharmacological effects and quality control of Shuxuening injection.

Free radical spiking tests were the primary approaches that were applied to measuring the antioxidative activity of TCMs and their preparations using spectrophotometry^31–33^. The free radicals mainly included DPPH or 2,2-azinobis- (3-ethyl benzothiazoline-6-sulfonic acid) (ABTS^+^). Most of the methods employing ABTS^+^ for measuring the total antioxidative activity of TCMs were off-line mode^8,34–36^. A novel cerium oxide nanoparticles-based colorimetric sensor using tetramethyl benzidine reagent was developed for antioxidant activity assay^37^. This novel method was more sensitive and selective than conventional spectrophotometry. In those experiments, however, the operations of these ways including off-line reaction, separation and detection were complicatedly step-by-step or some of them were even merely used for measuring total antioxidative activity. To simplify the steps, the on-line LC-ABTS^+^ methods^38–40^ for determining the antioxidative activity of TCMs were developed successively. Nevertheless, large amounts of organic reagent were needed in the whole experiment. In order to solve the existing problems, we developed an on-line method by integrating CE and ABTS^+^ for rapidly screening antioxidants from herbal medicine preparation and chose SI as the example to demonstrate the feasibility of this method.

As far as we know, the on-line ABTS^+^-CE-DAD method for screening antioxidant from herbal medicine has never been reported. Various ingredients with antioxidative property in herbal medicine were identified and the total antioxidative activity of herbal medicine was measured rapidly by on-line ABTS^+^-CE-DAD method. Not only contents of antioxidants but also the activity of sample was determined. Therefore, the quality of herbal medicine can be evaluated by both of them. In the present study, the precision and reliability of this on-line ABTS^+^-CE-DAD method were discussed. The on-line ABTS^+^-CE-DAD method was established to screen and quantify antioxidants from SI as well as monitor the quality. This activity-integrated ABTS^+^-CE-DAD method is a powerful tool for separation, screening and identifying the antioxidant components and for evaluating the quality of herbal medicines.

## Results and Discussion

### Optimization of on-line ABTS^+^-CE-DAD method

At the initial stage of experiment, the conditions including pH and concentration of buffer, the concentration of SDS, β-CD, ACN as well as voltage and temperature were optimized. These factors were the main ones influencing investigation. In several papers on study of antioxidant activity using CE and DPPH published before^24–26^, the optimized parameters were similar to the ones described here. Hence, the parameters chose to explore for the optimum performance were comprehensive and necessary.

#### Effect of buffer pH

**Figure 1:**
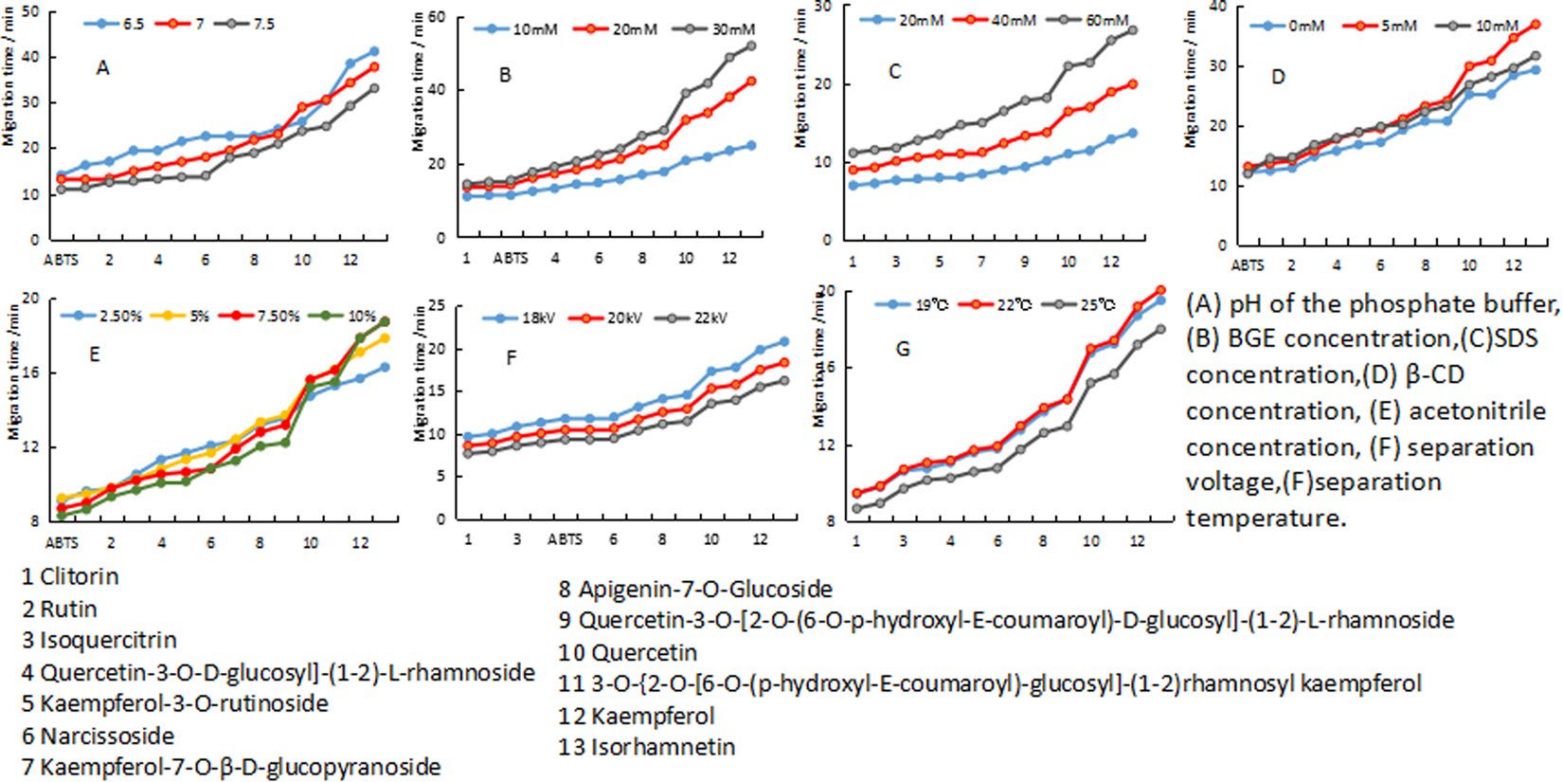
Effects of parameters on the migration time and resolution of fourteen (including ABTS^+^) peaks (n = 3).

For the separation performance including resolution and migration time, pH is the most critical factor^41,42^. In order to determine the optimal pH of BGE, different values of pH (6.5, 7.0 and 7.5) were investigated. As seen in Fig. 1(A), the migration time minimized when pH increasing from 6.5 to 7.5. The compounds didn’t have a good resolution at pH 6.5. In Fig. 1(A), the red and gray trend lines presented that the first several compounds were separated worse at pH 7.5 than pH 7.0. There is a slight difference between pH 7 and pH 7.5 in the total analyzed time. Therefore, pH 7 was selected to perform the following study.

#### Effect of buffer concentration

The different concentration of buffer causes different migration and resolution of ingredients of sample, so that the concentration of buffer needs to be investigated. Here, 10 mM, 20 mM and 30 mM of phosphate (pH 7.0) were observed. The Fig. 1(B) suggested that the migration time extended with increasing of the BGE concentration due to the decrease of EOF^43^. The almost flat blue line (10 mM) showed that separations of all compounds are not good. The highest grey line (30 mM) represented the longest migration time and the flat line between 2 and ABTS showed poor separation between rutin and ABTS. The optimized resolutions were obtained at 20 mM. Thus, 20 mM phosphate was selected as the optimum concentration of buffer.

#### Effect of SDS concentration

SDS is a common additive which can form micelles over its value of CMC and it plays an important role in Micellar capillary electrophoresis (MEKC). Hence, optimizing the concentration of SDS to obtain a good resolution is quite necessary. The effect of different concentrations (20 mM, 40 mM and 60 mM) on separation of multiple components in SI was investigated. As shown in Fig. 1(C), the analysis time was prolonged and the resolution became higher when SDS concentration increased from 20 mM to 60 mM. There was little difference of among compounds in y-axis, that is to say, the most of the ingredients of SI were not separated from each other at the 20 mM. At 60 mM, however, the resolution of narcissoside and kaempferol-7-O-β-D-glucopyranoside decreased than the concentration of 40 mM. Finally, the 40 mM of SDS was selected as the most suitable concentration.

#### Effect of β-CD concentration

CD with a special structure of cone-shaped cavity can improve the separation of similar molecules by altering their migration behavior^44–48^. In order to perfect the peak shape and improve the separation selectivity, β-CD was added into BGE solution. Keeping other conditions constant, the concentration of β-CD ranged from 0 mM to 10 mM. The analyzed time minimized and the width of peaks narrowed as the concentration of β-CD increased. Nevertheless, the resolution of peaks also decreased with increasing of β-CD and we can observe this phenomenon by comparing the three trend lines in Fig. 1(D). Taking various factors into consideration, 5 mM β-CD was the best concentration for separating and determining antioxidants.

#### Effect of acetonitrile (ACN) concentration

Most of BGE solutions are hydrosoluble and quite a number of samples cannot dissolve very well in such BGE. ACN as one of the frequently-used organic solutions was mixed into BGE to avoid the precipitation of buffer during the electrophoretic operation^43^. Besides, ACN has low UV absorption and low viscosity; hence it doesn’t significantly interfere with the detection of samples. The concentrations of ACN ranging from 2.5% to 10% were studied. As shown in the Fig. 1(E), the resolution of ingredients was not good at 2.5%. In spite of the increase of resolution, the analyzed time became longer with the concentration increasing. The shapes of peaks were better at 7.5% than those at 5%. Finally, 7.5% was used for further research.

#### Effect of voltage and temperature

Apart from those factors described above, voltage and temperature also influence the migration behavior of analytes. Voltage provides driving force, so that high voltage reduces running time and simultaneously creates more joule heat. Temperature is the major factor that influences the viscosity of BGE. Voltage (18 kV, 22 kV and 25 kV) and cassette temperature (19 °C, 22 °C and 25 °C) on the separation of compounds were examined. From Fig. 1(F and G), we can see that running time and resolution decreased with voltage and temperature increasing. When 22 kV was utilized, the best resolution was observed. The suitable separations for compounds were obtained at 22 °C.

Eventually, the optimal conditions of separating and screening antioxidants from SI was determined to be 20 mM phosphate buffer (pH 7.0), 40 mM SDS, 5 mM β-CD, 7.5% ACN, 22 kV and 22 °C.

### Screening of antioxidant of SI

**Figure 2:**
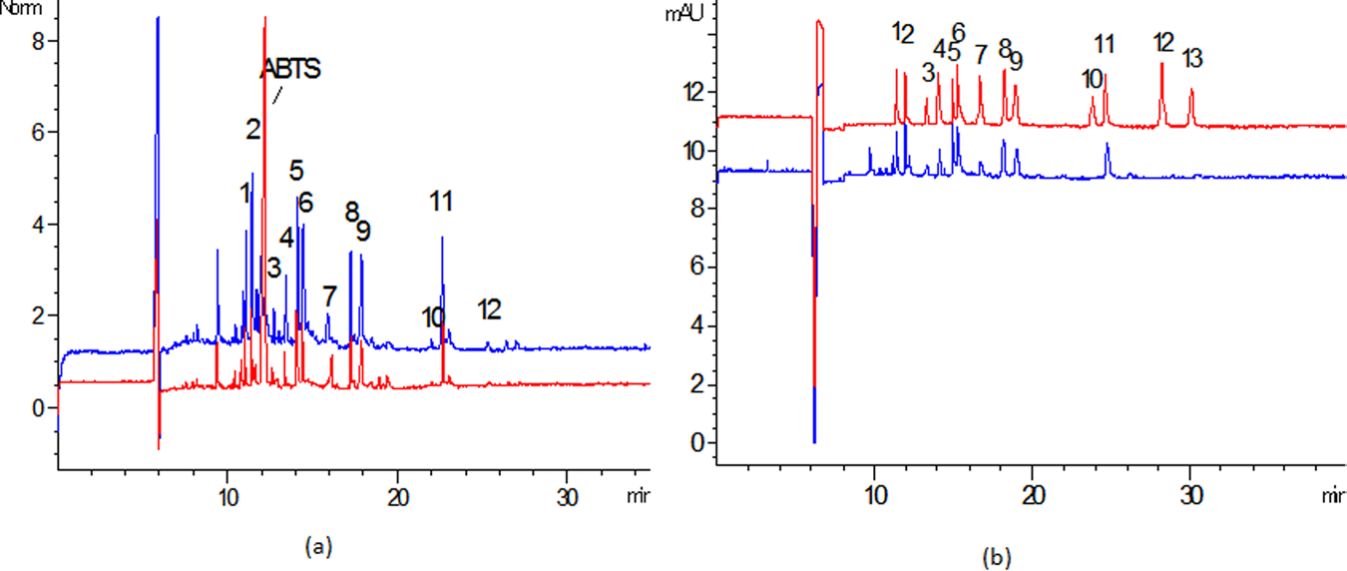
(**a**) Capillary electropherograms of SI: SI on-line mixed with H_2_O and on-line mixed with ABTS^+^ (n = 3). The blue one is the electrophoretogram of mixtrue of H_2_O and SI, and the red one is the electrophoretogram of mixture of ABTS^+^ and SI. (**b**) Capillary electropherograms of standard mixture of thirteen compounds and SI (n = 3). The blue one is the electrophoretogram of SI, and the red one is the electrophoretogram of standards (1 Clitorin, 2 Rutin,3 Isoquercitrin, 4 Quercetin-3-O-D-glucosyl]-(1-2)-L-rhamnoside,5 Kaempferol-3-O-rutinoside,6 Narcissoside,7 Kaempferol-7-O-β-D-glucopyranoside,8 Apigenin-7-O-Glucoside, 9 Quercetin-3-O-[2-O-(6-O-p-hydroxyl-E-coumaroyl)-D-glucosyl]-(1-2)-L-rhamnoside, 10 Quercetin,11, 3-O-{2-O-[6-O-(p-hydroxyl-E-coumaroyl)-glucosyl]-(1-2)rhamnosyl kaempferol, 12 Kaempferol,13 Isorhamnetin).

In order to screen antioxidants existing in SI, the ABTS^+^ (1.5 mg·mL^−1^) and SI diluted 2 times were successively injected into CE at 50 mbar pressure for 5 s. This detection was performed no less than three times. By comparison with the electrophoretogram of the sample without ABTS^+^, nine ingredients with antioxidative activity were screened from SI according to the decreased peak area (Fig. 2(a)**)**. Compared with the electrophoretogram of reference standards, those compounds were identified as clitorin, rutin, isoquercitrin, quercetin-3-O-D-glucosyl]-(1-2)-L-rhamnoside, kaempferol-3-O-rutinoside, Kaempferol-7-O-β-D-glucopyranoside, Apigenin-7-O-Glucoside, Quercetin-3- O-[2-O-(6-O-p-hydroxyl-E-coumaroyl)-D-glucosyl]-(1-2)-L-rhamnoside, 3-O-{2-O-[6-O-(p-hydroxyl-E-coumaroyl)-glucosyl]-(1-2)}rhamnosyl kaempferol (Fig. 2(b)). Judging from the results obtained above, those screened ingredients were the major constituents with antioxidative activity.

### Method validation

The calibration curves, linearity ranges of clitorin, rutin, isoquercitrin, quercetin-3-O-D-glucosyl]-(1-2)-L-rhamnoside, kaempferol-3-O-rutinoside, kaempferol-7-O-β -D-glucopyranoside, apigenin-7-O-Glucoside, quercetin-3-O-[2-O-(6-O-p-hydroxyl-E-coumaroyl) -D-glucosyl]-(1-2)-L-rhamnoside, 3-O-{2-O-[6-O-(p-hydroxyl-E-coumaroyl)-glucosyl]-(1-2) rhamnosyl kaempferol are listed in Table 1. The MDLs of the compounds ranged from 0.04 µg·mL^−1^ to 0.12 µg·mL^−1^, while MQLs ranged from 0.1 µg·mL^−1^ to 0.4 µg·mL^−1^.

**Table 1:** The calibration curves, linearity ranges, LODs, LOQs and recoveries of nine compounds (*P* = 95%, *k* = 2).

Compounds	Regression equation	R^2^	Linearity range(µg/ml)	MDL (µg/ml)	MQL (µg/ml)	Recovery	
						Means ± U(%)	RSD(%)
Clitorin	y = 0.2674 × − 0.0154	0.9999	4–128	0.09	0.3	100.8 ± 0.5	3.12
Rutin	y = 0.3479 × + 0.0736	0.9997	4–128	0.08	0.2	98.1 ± 0.9	3.91
Isoquercitrin	y = 0.4362 × − 0.0876	0.9996	1.5–48	0.04	0.1	104.5 ± 0.1	1.32
Quercetin-3-O-D-glucosyl]-(1-2)-L-rhamnoside	y = 0.3458 × − 0.3234	0.9994	5–160	0.07	0.2	98.9 ± 0.6	3.76
Kaempferol-3-O-rutinoside	y = 0.4151 × − 0.0989	0.9994	2.5–120	0.12	0.4	99.8 ± 1.0	3.46
Kaempferol-7-O-β-D-glucopyranoside	y = 0.7537 × − 0.8358	0.9998	2.5–80	0.04	0.1	100.7 ± 0.3	4.49
Apigenin-7-O-Glucoside	y = 0.719 × − 0.3239	0.9997	2.5–80	0.07	0.2	100.2 ± 0.8	0.95
Quercetin-3-O-[2-O-(6-O-p-hydroxyl-E-coumaroyl)-D-glucosyl]-(1-2)-L-rhamnoside	y = 0.3604 × − 0.2522	0.9999	6–192	0.09	0.3	96.9 ± 0.3	0.79
3-O-{2-O-[6-O-(p-hydroxyl-E-coumaroyl)-glucosyl]-(1-2)rhamnosyl kaempferol	y = 0.5507 × − 0.3791	0.9998	4–128	0.05	0.1	103.3 ± 0.5	1.04

In order to validate the reliability of this method, the precision and accuracy including intra- and inter-day were evaluated and the results were showed in Table 2. The results of precision of each compound varied slightly and the RSDs of ABTS^+^ were 2.04% and 3.35%, respectively. Accuracy of ingredients was in the range of 96.7–107%. Compared with acceptability criteria, these results were acceptable indicating that this method was reliable, accurate and repeatable.

**Table 2:** Intra-day and Inter-day accuracy and precision, stability of nine compounds (*P* = 95%, *k* = 2).

Compounds	Ca (µg/mL)	Intraday		Interday		Stability for 24 h	
		Accuracy(%)	Cd (µg/mL)	Accuracy(%)	Cd (µg/mL)	Remains (%)	Cd (µg/mL)
Clitorin	12	100	12.0 ± 0.9	101	12.1 ± 0.8	101	12.0 ± 0.9
	48	99.6	47.8 ± 3.1	100	48.1 ± 2.1	100	47.8 ± 2.2
	96	101	97.0 ± 2.7	101	97.0 ± 4.4	101	97.6 ± 4.3
Rutin	12	96.7	11.6 ± 1.0	97.5	11.7 ± 1.3	98.3	11.6 ± 1.4
	48	100	48.1 ± 2.1	100	48.0 ± 1.7	99.3	47.9 ± 1.6
	96	101	97.0 ± 3.3	100	96.2 ± 3.8	101	97.0 ± 2.4
Isoquercitrin	4.5	97.8	4.4 ± 0.3	97.8	4.4 ± 0.5	96.6	4.3 ± 0.5
	18	102	18.3 ± 1.0	100	18.1 ± 1.0	97.0	18.0 ± 1.0
	36	107	38.4 ± 1.8	102	36.7 ± 3.4	99.0	37.4 ± 2.8
Quercetin-3-O-D-glucosyl]-(1-2)-L-rhamnoside	15	98.7	14.8 ± 1.0	98.0	14.7 ± 0.9	99.4	14.7 ± 1.0
	60	101	60.6 ± 3.9	100	60.1 ± 3.5	96.8	59.6 ± 3.7
	120	106	127.6 ± 4.4	102	121.8 ± 9.7	98.7	123.9 ± 8.3
Kaempferol-3-O-rutinoside	7.5	101	7.6 ± 1.1	98.7	7.4 ± 0.8	97.0	7.5 ± 1.0
	30	102	30.7 ± 1.8	102	30.6 ± 1.7	97.8	30.4 ± 1.5
	60	107	64.1 ± 2.9	102	61.4 ± 3.4	99.1	62.1 ± 4.8
Kaempferol-7-O-β-D-glucopyranoside	7.5	97.3	7.3 ± 1.1	98.7	7.4 ± 0.6	100	7.3 ± 0.8
	30	105	31.4 ± 1.9	102	30.6 ± 1.7	96.2	30.8 ± 1.82
	60	106	63.5 ± 2.0	102	61.4 ± 3.4	98.4	62.0 ± 3.5
Apigenin-7-O-Glucoside	7.5	98.7	7.4 ± 0.7	98.7	7.4 ± 0.4	98.9	7.4 ± 0.5
	30	99.7	29.9 ± 0.5	100	30.0 ± 0.9	99.2	29.8 ± 0.8
	60	104	62.1 ± 2.5	100	60.3 ± 3.6	99.6	61.1 ± 2.8
Quercetin-3-O-[2-O-(6-O-p-hydroxyl-E-coumaroyl)-D-glucosyl]-(1-2)-L-rhamnoside	18	100	18.0 ± 1.2	98.9	17.8 ± 0.9	97.2	17.8 ± 1.0
	72	101	72.8 ± 3.9	100	72.1 ± 2.9	97.6	72.0 ± 3.5
	144	102	147.3 ± 2.9	101	145.4 ± 4.0	99.4	145.9 ± 3.6
3-O-{2-O-[6-O-(p-hydroxyl-E-coumaroyl)-glucosyl]-(1-2)rhamnosyl kaempferol	12	96.7	11.6 ± 0.7	98.3	11.8 ± 0.7	100	11.7 ± 0.7
	48	99.6	47.8 ± 1.6	100	48.1 ± 1.4	100	47.9 ± 1.4
	96	102	97.8 ± 0.8	101	96.7 ± 2.4	99.4	97.0 ± 2.0

–Ca is the actual concentration and Cd is the detected concentration.

Another factor investigated was the stability for 24 h. This was conducted in order to evaluate the stability of the analyzed compounds in sample. The results were listed in the Table 2, showing that the remains of nine target compounds preserved at 4 °C for 24 h were in the range of 96.6–101%, 96.2–100% and 98.4–101% at three levels and all of the detected concentrations were close to accurate value. Additionally, remain of ABTS^+^ was 99.6% with 3.76% of RSD. These results showed that the determined compounds were stable during sample storage and running in CE apparatus.

The tested recoveries are given in the Table 1. Obviously, the average recoveries of nine active antioxidants were within 96.9–104.5% and RSDs were less than 5.96%. Therefore, the method for screening and quantifying antioxidants in TCMs injection was stable, precise and applicable.

### Contents of nine antioxidants in sample

Using the initially developed approach, the contents of nine antioxidants in 20 batches of SI were detected, respectively (Table 3**)**. The concentration range of clitorin, rutin, isoquercitrin, quercetin-3-O-D-glucosyl]-(1-2)-L-rhamnoside, kaempferol-3-O-rutinoside, kaempferol-7-O- β-D-glucopyranoside, apigenin-7-O-Glucoside, quercetin-3-O-[2-O-(6-O-p-hydroxyl-E- coumaroyl)-D-glucosyl]-(1-2)-L-rhamnoside, 3-O-{2-O-[6-O-(p-hydroxyl-E-coumaroyl)-glucosyl] -(1-2)rhamnosyl kaempferol were determined as 51.7–66.9 µg·mL^−1^, 58.6–76.7 µg·mL^−1^, 12.2–18.1 µg·mL^−1^, 35.7–57.8 µg·mL^−1^, 57.9–92.5 µg·mL^−1^, 7.0–10.3 µg·mL^−1^, 30.5–42.3 µg·mL^−1^, 65.5–86.9 µg·mL^−1^ and 45.2–83.1 µg·mL^−1^, respectively. The average of total amount of 20 batches was further calculated and the result was 0.453 mg·mL^−1^ with 7.21% of RSD%. These results presented the fact that there was a slight difference among 20 batches. This phenomenon was in accordance with the result obtained above that different batch had different antioxidative activity.

**Table 3:** Contents of nine compounds and total inhibition rate of different samples and the result of (µg/mL) (*P* = 95%, *k* = 2).

Sample	1	2	3	4	5	6	7	8	9	10
Clitorin	58.8 ± 2.1	58.4 ± 2.3	62.6 ± 1.5	61.7 ± 0.4	62.3 ± 2.5	61.5 ± 0.42	60.5 ± 1.9	63.8 ± 1.3	53.8 ± 0.4	51.7 ± 2.2
Rutin	69.4 ± 6.1	67.1 ± 1.3	67.4 ± 3.4	69.7 ± 1.8	58.6 ± 3.0	68.6 ± 5.1	66.0 ± 14.5	69.8 ± 10.8	72.0 ± 2.9	59.3 ± 3.0
Isoquercitrin	18.1 ± 0.4	16.5 ± 1.1	16.6 ± 2.1	16.1 ± 0.3	17.2 ± 0.4	16.0 ± 1.1	17.0 ± 0.4	18.4 ± 0.7	17.5 ± 0.7	12.7 ± 1.1
Quercetin-3-O-D-glucosyl]-(1-2)-L-rhamnoside	45.2 ± 1.8	43.5 ± 2.1	45.7 ± 3.5	45.2 ± 1.0	44.3 ± 1.4	44.1 ± 2.1	42.6 ± 1.2	45.3 ± 1.8	39.5 ± 0.3	35.7 ± 0.7
Kaempferol-3-O-rutinoside	87.4 ± 1.6	86.7 ± 1.2	92.5 ± 2.0	88.2 ± 0.3	87.7 ± 0.9	87.0 ± 1.2	88.0 ± 1.5	90.8 ± 0.8	81.2 ± 1.3	73.6 ± 2.3
Kaempferol-7-O-β-D-glucopyranoside	10.0 ± 0.3	9.6 ± 0.9	9.8 ± 1.2	9.1 ± 0.3	10.1 ± 0.3	9.6 ± 0.8	8.8 ± 1.3	10.1 ± 0.4	8.8 ± 0.2	9.8 ± 1.5
Apigenin-7-O-Glucoside	37.9 ± 1.4	37.9 ± 0.2	40 ± 1.3	42.3 ± 1.6	39.3 ± 0.6	38.2 ± 2.8	39.0 ± 0.7	41.4 ± 1.1	34.8 ± 0.4	33.9 ± 1.1
Quercetin-3-O-[2-O-(6-O-p-hydroxyl-E-coumaroyl)-D-glucosyl]-(1-2)-L-rhamnoside	86.9 ± 2.5	83.8 ± 2.3	84.4 ± 4.7	82.5 ± 3.8	80.4 ± 0.9	80.4 ± 5.5	80.4 ± 1.4	85.1 ± 3.5	79.0 ± 0.8	77.8 ± 2.2
3-O-{2-O-[6-O-(p-hydroxyl-E-coumaroyl)-glucosyl]-(1-2)rhamnosyl kaempferol	74.3 ± 1.1	75.6 ± 0.8	81.3 ± 3.0	79.8 ± 3.6	82.1 ± 1.8	76.4 ± 2.2	78.4 ± 1.8	83.1 ± 5.0	58.2 ± 2.9	54.8 ± 0.2
Inhibition(%)	34.1	30.2	36.7	35.0	33.7	33.2	31.9	35.2	29.9	21.7
**Sample**	**11**	**12**	**13**	**14**	**15**	**16**	**17**	**18**	**19**	**20**
Clitorin	51.9 ± 2.2	62.1 ± 0.7	59.0 ± 0.4	60.6 ± 0.0	64.9 ± 3.0	66.9 ± 1.9	61.4 ± 2.0	61.8 ± 1.3	61.6 ± 2.6	63.4 ± 1.6
Rutin	65.3 ± 1.1	74.0 ± 1.5	69.3 ± 2.4	74.2 ± 0.9	76.7 ± 1.8	63.2 ± 16.1	73.1 ± 0.6	75.4 ± 2.1	75.8 ± 2.9	76.2 ± 4.1
Isoquercitrin	12.6 ± 0.3	13.4 ± 2.1	12.2 ± 0.3	12.8 ± 0.0	15.5 ± 0.5	13.4 ± 2.3	13.6 ± 1.0	17.3 ± 1.3	16.5 ± 0.3	17.0 ± 1.0
Quercetin-3-O-D-glucosyl]-(1-2)-L-rhamnoside	35.9 ± 0.6	39.8 ± 1.2	36.3 ± 0.7	57.8 ± 8.8	39.4 ± 1.0	39.4 ± 3.8	40.3 ± 2.1	40.1 ± 1.2	38.3 ± 0.9	38.9 ± 0.9
Kaempferol-3-O-rutinoside	74.2 ± 0.5	81.6 ± 0.7	78.7 ± 1.0	57.9 ± 7.8	77.5 ± 2.4	89.9 ± 2.4	83.8 ± 1.8	84.7 ± 2.2	85.8 ± 2.7	87.6 ± 1.8
Kaempferol-7-O-β-D-glucopyranoside	9.1 ± 0.6	7.8 ± 0.6	8.7 ± 1.2	7.2 ± 0.7	8.7 ± 0.0	9.2 ± 1.3	10.3 ± 0.7	7.6 ± 0.6	7.1 ± 0.6	7.0 ± 0.0
Apigenin-7-O-Glucoside	33.8 ± 1.5	33.6 ± 1.7	32.8 ± 0.4	33.6 ± 2.2	30.9 ± 2.7	31.8 ± 3.8	30.5 ± 2.2	33.0 ± 1.1	32.8 ± 1.5	34.1 ± 0.7
Quercetin-3-O-[2-O-(6-O-p-hydroxyl-E-coumaroyl)-D-glucosyl]-(1-2)-L-rhamnoside	77.0 ± 2.8	68.1 ± 2.0	65.5 ± 4.2	71.5 ± 4.2	71.2 ± 7.0	71.5 ± 4.7	68.6 ± 0.6	66.5 ± 1.4	68.1 ± 1.9	71.2 ± 0.6
3-O-{2-O-[6-O-(p-hydroxyl-E-coumaroyl)-glucosyl]-(1-2)rhamnosyl kaempferol	54.2 ± 0.8	48.6 ± 1.0	45.2 ± 1.0	50.3 ± 1.5	47.4 ± 1.5	55.0 ± 1.6	49.0 ± 0.2	45.9 ± 0.4	45.8 ± 1.6	48.5 ± 0.4
Inhibition(%)	25.1	26.4	22.5	26.2	26.9	27.1	26.9	26.9	26.1	28.1

### On-line determination of total antioxidative activity of samples

**Figure 3:**
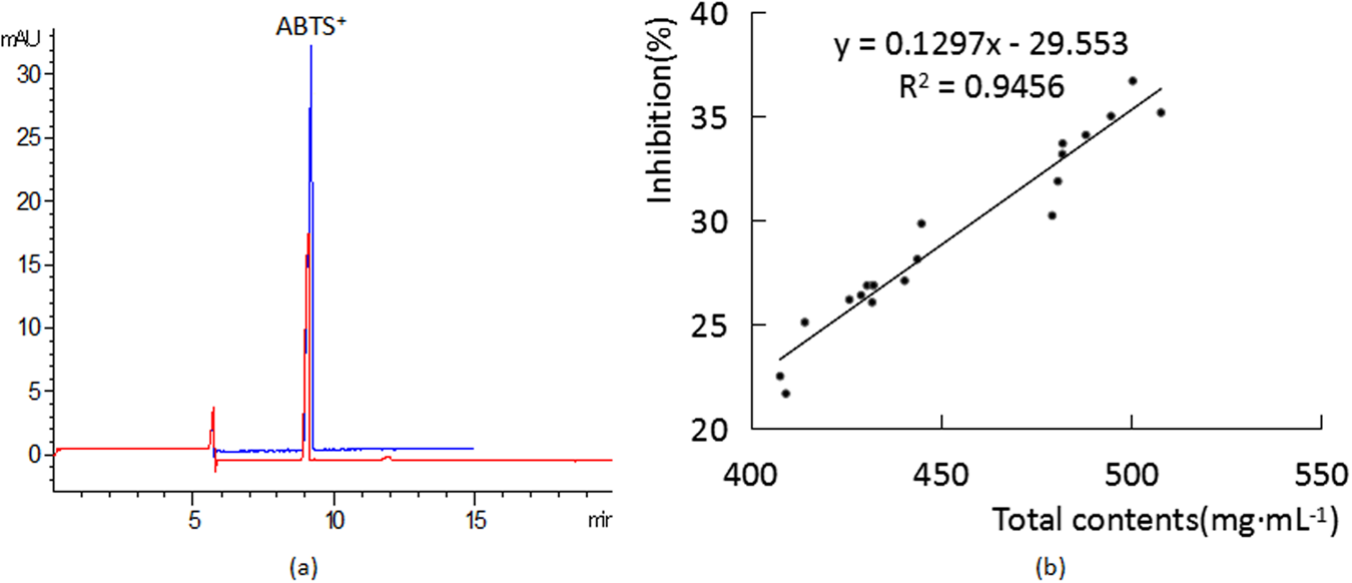
(**a**) Capillary electropherograms of ABTS^+^: ABTS^+^ solution; on-line mixed with SI (n = 3). The blue one is the electrophoretogram of ABTS^+,^ and the red one is the electrophoretogram of mixture. (**b**) Relationship of the total quantitative and antioxidant activity of SI.

The total antioxidative activity of samples was also determined by on-line ABTS^+^-CE-DAD. Considering that ABTS^+^ is scavenged easily by SI, all of the samples were diluted 32 times using deionized water before injection. Then, 1.5 mg·mL^−1^ of ABTS^+^ was on-line mixed with diluted sample or equivalent water separately. This test was performed in triplicate. According to obtained electrophoretograms in Fig. 3(a), the peak area of ABTS^+^ was reduced. Comparing the two absorbance of ABTS^+^, the relative percentage of inhibition for ABTS^+^ was calculated with the following equation (Inhibition (%) = (P_0_ − P_1_)/P_0_ × 100%) where P_0_ was the peak area of ABTS^+^ on-line mixing with water and P_1_ was the peak area of ABTS^+^ mixing with diluted sample. The results of all 20 batches of SI are summarized in Table 3 and those data proved that SI has antioxidative activity. Moreover, the minor difference of activity existed in different batch. Therefore, this established on-line ABTS^+^-CE-DAD method can be used to evaluate the total antioxidative activity of preparations of TCMs with complex matrix.

In our experiment, the relationship between total content and total antioxidative activity was studied with the purpose of evaluating the quality of SI. As is shown in Fig. 3(b), the antioxidative activity of SI has high correlation with the total amounts of the nine antioxidants (R^2^ = 0.9456). This result indicates that the nine ingredients can be selected as combinatorial active markers to assess the quality of SI and that this on-line ABTS^+^-CE-DAD method can be utilized for assessing the antioxidative activity of SI among different batches.

### Comparison with other antioxidant activity assay methods

Compared the characteristic of this developed method with the ones on antioxidant activity assay published before, advantages and drawbacks of them were summarized in Table 4. Obviously, the total antioxidative activity can measured but the antioxidants cannot be screened when off-line mode is used. Although on-line HPLC-UV-ABTS^+^ can screen the antioxidants from samples with complex matrix, a large amount of solvent against the environment is consumed. The on-line DPPH-CE-DAD is similar to this developed method, but ABTS assay is more effective for the analysis of antioxidants due to the faster reaction kinetics with ABTS^+^^40^. Therefore, this developed on-line ABTS^+^-CE-DAD method is reliable, simple, environmentally-friendly for simultaneously measuring antioxidative activity and screening antioxidants from samples.

**Table 4:** Comparison of this proposed methods with other antioxidant activity assay.

Analysis samples	Analysis modes	Detection methods	Solvent volume (mL)	Reaction time (min)	Separation of antioxidants	References
Polysaccharides of *potato peels*	ABTS^+^ -offline	Spectrophotometer	3	6	×	^32^
A plant extract	Colorimetric sensor of cerium oxide nanoparticles	Spectrophotometer	4	0	×	^37^
Antioxidant components of *Viburnum opulus L*.	On-line HPLC-UV-ABTS^+^	HPLC	78	0.1	√	^40^
Reduning injection	On-Line DPPH-CE-DAD	CE	0.042	0	√	^24^
Shuxuening injection	On-line ABTS^+^-CE-DAD	CE	0.084	0	√	This developed method

## Conclusions

ABTS^+^ is one of frequently-used free radical for evaluating the antioxidative activity of herbal medicine and chemical components. The simple and reliable on-line ABTS^+^-CE-DAD method was developed and validated to screen the antioxidants from herbal medicines. ABTS^+^ was firstly on-line integrated with CE-DAD method to develop to quantify multiple antioxidants for evaluating the quality of herbal medicines. Nine major antioxidants were screened and selected as combinatorial active markers to evaluate quality control of Shuxuening injection. Compared with traditional methods, on-line ABTS^+^-CE-DAD method can be applied to evaluating the quality of TCMs preparations combining their pharmacological activity based on holistic view. In addition, it consumes less organic reagents. It was demonstrated that on-line ABTS^+^-CE-DAD method was eco-friendly powerful technique to screen and quantify active ingredients for evaluating quality of herbal medicines. Moreover, this proposed method also can be used for rapidly measuring antioxidant activity of sample with unknown ingredients and investigating antioxidant activity of herbal medicine and its preparations. In the future, on-line ABTS^+^-CE-DAD will be combined with the mass spectrum (MS) to determine unknown antioxidants and combined with other enrichment techniques to determine the trace antioxidants in herbal medicines.

## Materials and Methods

### Chemicals and Reagents

Ten reference compounds including clitorin, rutinum, isoquercitin, quercetin-3-O-D-glucosyl]-(1-2)-L-rhamnoside, kaempferol 3-rutinoside, kaempferol-7-O-β-D- glucopyranoside, apigenin-7-O-Glucoside, quercetin-3-O-[2-O-(6-O-p-hydroxyl-E-couMaroyl) -D-glucosyl]-(1-2)-L-rhaMnoside, quercetin, 3-O-{2-O-[6-O-(p-hydroxyl-E-coumaroyl)-glucosyl]-(1-2)rhamnosyl} kaempferol were purchased from Chengdu Must Bio. Sci. and Tec. Co. Ltd. (Chengdu, China). 20 batches of Shuxuening injection were obtained from Shineway Pharmaceutical Co. Ltd. (Shijiazhuang, China). A Milli-Q Academic ultra-pure water system purchased from Milford (Millipore, USA) supplied deionized water which was utilized for the preparation of sample and buffer solution. Acetonitrile (ACN) and methanol (MeOH) were from Merck (Chromatographic, Germany) and ABTS and potassium persulfate were from Sigma (Analytical, USA). The other chemicals were analytical reagents.

### Instrumentation and conditions

All experiments were performed on an Agilent 7100 CE system equipped with a Diode Array Detector (Waldbronn, Germany). Agilent ChemStation software was used to control the instrument and analyze the resulting data. The CE separations were performed on a fused silica capillary with a dimension of 50 µm I.D. and a total length of 60.2 cm (effective length of 52 cm) (Ruifeng, Hebei, China). A new capillary was pretreated by flushing sequentially with 1.0 M NaOH, 0.1 M NaOH and deionized water under a pressure of 940 mbar for 10 min each. Prior to every run, the capillary was rinsed with 0.1 M NaOH for 2 min and then deionized water for 2 min followed by the background electrolyte (BGE) for 3 min at a pressure of 940 mbar. After the last separation of each day, capillaries were washed orderly with 0.1 M NaOH and deionized water for 10 min, respectively. BGEs in vials need exchange for every two runs in order to obtain the highest reproducibility of the migration times.

### The condition of method

**Figure 4:**
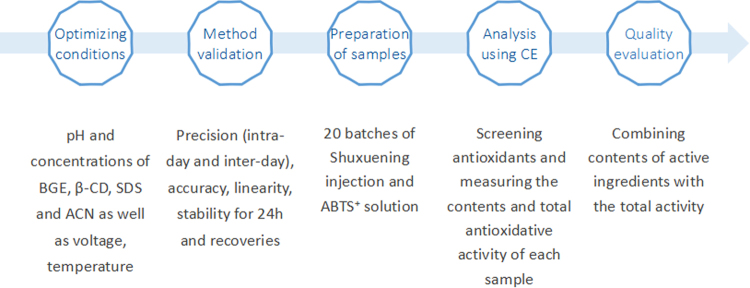
The diagram of the steps of the experiment procedure.

The running buffer for determining and on-line screening antioxidants was composed of 20 mM phosphate (pH 7.0), 5 mM β-cyclodextrin (β-CD), 40 mM sodium dodecyl sulfate (SDS) and 7.5% ACN. The phosphate with pH 7.0 was obtained by mingling NaH_2_PO_4_ solution with equal concentration Na_2_HPO_4_ at an appropriate ratio. A voltage of 20 kV was applied and the temperature of the capillary cartridge maintained at 22 °C in the whole process. All samples were injected into CE instrument for 5 s under 50 mbar. The wavelengths for detecting antioxidants and ABTS^+^ are 360 nm and 405 nm, respectively. The steps of the experiment procedure were showed in Fig. 4.

### Preparation of standard solutions and samples

All flavonoid standards were individually dissolved with MeOH and then the mixed standards as stock solution were dissolved to 0.1 mg·mL^−1^ in 50% MeOH. ABTS and potassium persulfate were prepared by deionized water. ABTS radical cation (ABTS^+^) was produced by mixing 300 µL of ABTS (9 mg·mL^−1^) and 600 µL of potassium persulfate (1.516 mg·mL^−1^). The mixture was kept in the dark at room temperature for 20–24 h to allow the thorough generation of radicals. The radical was stable in this system for 3–4 days in the dark at room temperature. The radical was then diluted with deionized water to 1.5 mg·mL^−1^ before use. Standard solutions were stored at 4 °C. Except for ABTS^+^. All solutions were filtered through a 0.22 mm nylon syringe filter prior to use.

### Preparation of Quality Control Samples

The low, medium and high concentration of Quality Control Samples of clitorin, rutinum, isoquercitin, quercetin-3-O-D-glucosyl]-(1–2)-L-rhamnoside, kaempferol 3-rutinoside, kaempferol-7-O-β-D-glucopyranoside, apigenin-7-O-Glucoside, quercetin-3-O-[2-O-(6-O-p-hydroxyl-E-couMaroyl)-D-glucosyl]-(1- 2)-L-rhaMnoside, quercetin, 3-O-{2-O-[6-O-(p-hydroxyl-E-coumaroyl)-glucosyl]-(1-2) rhamnosyl kaempferol were prepared by dissolving the specific mixed standard solution with different volumes of 50% MeOH.

### Method validation

Firstly, the conditions including pH and concentrations of BGE, β-CD, SDS and ACN as well as voltage, temperature were optimized to separate the antioxidants of SI and ABTS^+^. Then, SI was mixed with ABTS^+^ or deionized water on line, respectively. Since peaks of antioxidants would decrease during on-line reaction of SI with ABTS^+^, the antioxidants could be screened by comparing the two electrophoretograms. The total antioxidative property of SI was measured by comparison of the peaks of ABTS^+^ obtained by on-line mixing ABTS^+^ with dilute SI and deionized water respectively. During the analysis time, the SI and ABTS^+^ were injected by 50 mbar for 5 s.

The prerequisite for analyzing sample is the availability of this developed method. Thus, proper quality assurance/quality control (QA/QC) procedures were performed for controlling and validating proposed method, including method detection limit (MDL), method quantitation limit (MQL), linearity, precision, accuracy, stability and recovery. In addition, measurement uncertainty was also carried out for estimating data due to uncertainty exists in all the individual steps of a chromatographic analysis. MDL and MQL were determined by using a solution containing the analytes with a specific matrix, that is method limits which is a relative measure avoiding matrix influence. MDL and MQL were separately calculated by formulas MDL = 3 *s*/√*n* and MQL = 10 *s*/√*n*, which *s* is standard deviation of replicates and *n* is number of replicates^49^.

Calibration curves of analytes were established with six concentrations. Precision and stability for 24 h were evaluated by analyzing low, middle and high (n = 6) concentrations of mixed standard solution. The recoveries of nine standards were performed by adding equivalent amount of them into a certain amount of SI sample. Both of original and spiked sample were determined using the optimized condition mentioned above and the recovery was calculated by the formula: Recovery (%) = (Measured amount − original amount)/spiked amount × 100%. Due to the uncertainty of the result, precision and stability were showed as a confidence interval with the form of mean ± U (expanded uncertainty), and the expanded uncertainty were calculated according to the review reported before^50^. In general, the value of *r* above 0.990 suggested that calibration curves have good correlation and recoveries recommend ranging from 95% to 105%. The mean value of accuracy should be within 5% the actual value and acceptable value of precision should not exceed 5%^51^.
